# Symptomatic Pectus Excavatum in Seniors: An Exploratory Study on Clinical Presentation and Incidence in Daily Practice

**DOI:** 10.5402/2013/373059

**Published:** 2013-02-25

**Authors:** Ron A. G. Winkens, Frank I. Guldemond, Paul F. H. M. Hoppener, Hans A. Kragten, J. Andre Knottnerus

**Affiliations:** ^1^Department of Integrated Care, Maastricht University Medical Centre, P.O. Box 5800, 6202 AZ Maastricht, The Netherlands; ^2^Department of General Practice, Maastricht University, P.O. Box 616, 6200 MD Maastricht, The Netherlands; ^3^Department of Cardiology, Atrium Medical Centre, P.O. Box 4446, 6401 CX Heerlen, The Netherlands

## Abstract

*Objectives*. Doctors all over the world consider a pectus excavatum usually as an incidental finding. There is some evidence suggesting that a pectus excavatum may cause symptoms in the elderly. It is not known how often a pectus excavatum occurs and how strong the relation is with symptoms. *Methods*. In hospitals and general practice data, we searched for evidence of a connection between cardiac symptoms and the presence of a pectus excavatum in a retrospective survey among patients in whom a pectus excavatum was found in a chest X-ray. In radiology reports, we searched for “pectus excavat^∗^ ” in almost 160000 chest X-rays. The identified X-rays were reviewed by 2 radiologists. Reported symptoms were combined to a severity sum score and the relation with pectus excavatum was assessed through logistic regression. *Results*. Pectus excavatum was found in 1 to 2 per 1000 chest X-rays. In 32% of patients (*N* = 117), we found symptoms that might reflect the presence of symptomatic pectus excavatum. We found a significant relation between the SPES sum score and the radiological level of pectus excavatum. *Conclusions*. A pectus excavatum found when examining the patient should not be neglected and should be considered as a possible explanation for symptoms like dyspnoea, fatigue, or palpitations.

## 1. Introduction 

In patients with symptoms like dyspnoea or fatigue, it is unlikely that a pectus excavatum is considered as a possible cause. Doctors all over the world consider a pectus excavatum usually as an incidental finding during examination or in radiographic procedures without any clinical significance, despite the fact that there may be a considerable compression visible and relocation of thoracic organs.

**Figure 1:**
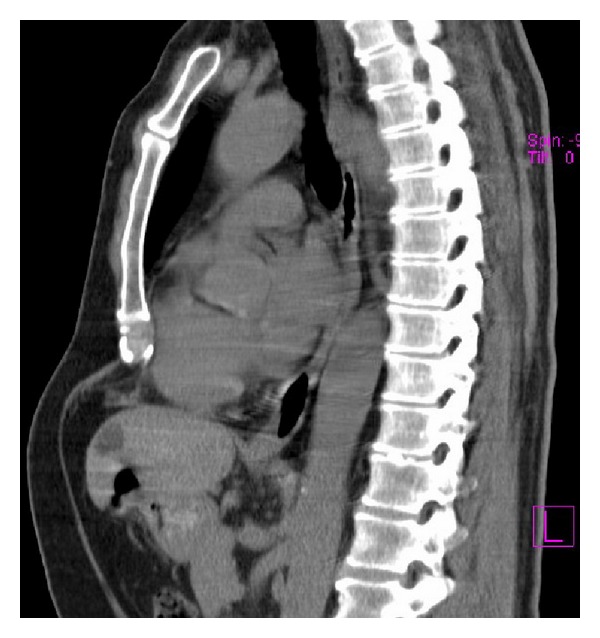
CT chest image of a male patient with PE.

Pectus excavatum is a deformity of the sternum and ribs caused by an unbalanced costochondral hypertrophy. It is a congenital abnormality, occurring in 1 per 300 to 1000 patients; it occurs mostly in boys and frequently within families [1–4]. The deformity of the chest wall may be noticed at birth but becomes more pronounced during adolescence (Figure 1).

Pectus excavatum is usually considered to be only a cosmetic problem, although severe cases may notice difficulties in breathing [1, 2].

There is some evidence suggesting that a pectus excavatum may cause symptoms. In the last decades, few publications have described symptomatic pectus excavatum and considered this as a possible cause of serious symptoms like shortness of breath, palpitations, chronic fatigue, and chest pain in elderly patients, leading to considerable physical impairment [2–6]. The clinical presentation of symptomatic pectus excavatum is described in Table 1.

Given the aforementioned clinical manifestation of symptomatic pectus excavatum in seniors (SPES), it bears all potentials to be a condition with a major impact on patient health and well-being. Unfortunately, it is not known how often symptomatic pectus excavatum in seniors may occur in daily practice and how strong the relation is between the presence of pectus excavatum and the possibly related symptoms. The literature on this issue is scarce and incidence data are absent. In the current paper, we, therefore, describe a retrospective survey of hospital and primary care data on the prevalence of pectus excavatum in radiological examinations and the degree in which symptoms are found in this subset of patients with pectus excavatum. The survey addressed the following questions.

1. How frequent is pectus excavatum seen and described as a finding at routine radiological examinations (in seniors)?
2. How often do elderly patients with confirmed pectus excavatum have symptoms suggesting the presence of symptomatic pectus excavatum in seniors?
3. Is there a relation between the severity of the pectus excavatum and the severity of symptoms?

## 2. Materials and Methods

We searched for evidence of a connection between cardiac symptoms and the presence of a pectus excavatum in a retrospective survey, using the following procedure.

The initial step was a text search in the (digital) reports of chest X-rays in two hospitals (the Maastricht University Medical Centre (MUMC) and the Atrium Medical Centre Heerlen), both located in the south of the province of Limburg, The Netherlands. We included cases in which the chest X-ray was ordered by a general practitioner (GP), cardiologist, or lung specialist in the years 2003–2006 and when a pectus excavatum (“pectus excavat*”) was reported. Patient age had to be between 50 and 75 years.

The selected chest X-rays were then rechecked by two radiologists (both blinded for the presence of symptoms) to confirm or exclude the pectus excavatum. The shape and degree of deformity (anteroposterior and left-right) of the chest wall were assessed and categorised (0: no pectus excavatum visible; 1: insignificant pectus excavatum, bowl-shaped; 2: minor pectus excavatum, round shape; 3: moderate pectus excavatum, round shape; 4: overt pectus excavatum, round shape, sometimes asymmetrical; 5: severe pectus excavatum, wedge shape, sometimes asymmetrical). In the literature, no other satisfying measure was found for pectus excavatum. Only one index might be relevant, the so-called Haller index, that is calculated by the following equation:

(1) $$\begin{array}{c} \frac{\text{maximal}\;\text{width}\;\text{of}\;\text{the}\;\text{thorax}}{\text{minimal}\;\text{depth}\;\text{of}\;\text{the}\;\text{thorax}\;\text{at}\;\text{the}\;\text{excavation}}. \end{array}$$

A Haller index above 3,25 is considered to reflect significant pectus excavatum [7, 8].

Retrospectively, clinicians were asked to check the hospital medical record system and they sent—if considered necessary—questionnaires to the GP of the selected patients to obtain information about the relevant medical history of each patient.

After that, we obtained anonymous data per patient. To assess to what extent patients suffer from symptoms, related to or caused by pectus excavatum, we categorised the signs, symptoms and possible test results from the patient's medical record or in the response of the GP, adding a severity score to each reported finding (see Table 2).

Next, we combined these to a sum score from 1 to 20. Any other preexisting known disorder that could be responsible for the symptoms was recorded and considered in the results. The SPES sum score was 0 in case of absence of a pectus excavatum or when symptoms were absent.

To assess the prevalence of pectus excavatum, we assessed how often a pectus excavatum was found in all chest X-rays in the two participating hospitals. We also calculated the percentage of patients with pectus excavatum who had cardiac or respiratory symptoms that could not be explained by any other disorder.

To determine a possible (causal) relation between pectus excavatum and symptoms, we compared the SPES severity sum score with the scores for the severity of pectus excavatum (the Haller index and our own judgement of severity) in a logistic regression analysis in which the (dichotomised) SPES sum score was the dependent variable. 

The Medical Ethics Committee of both the Maastricht University Medical Centre (MUMC) and the Atrium Medical Centre Heerlen approved the study. Informed consent of patients was not required.

## 3. Results

**Figure 2:**
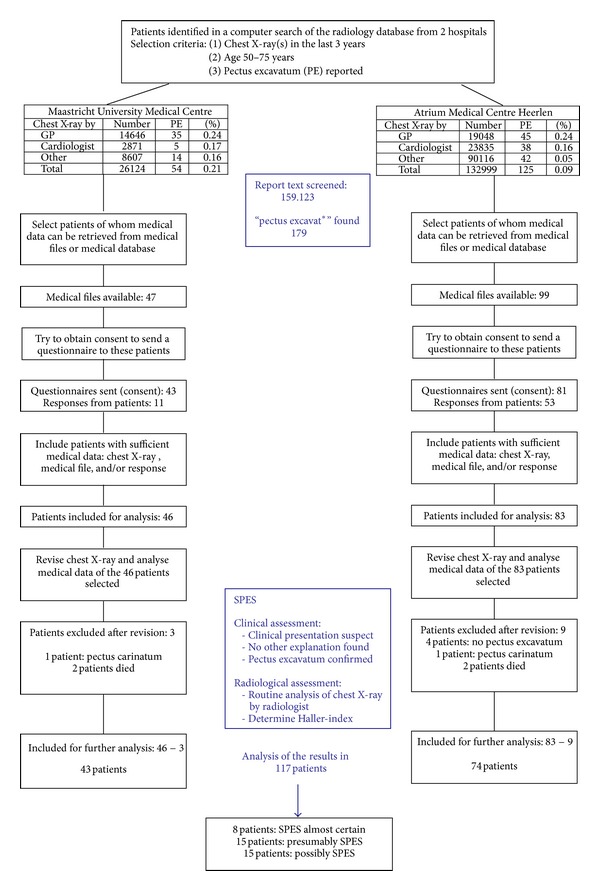
Flow chart patient selection and inclusion.

In the 159122 chest X-rays that we analysed, we found 179 patients with a pectus excavatum (Figure 2). Thus, our data revealed a prevalence of one per 889 patients, roughly between one per 1000 (Atrium MC Heerlen) and one per 500 (MUMC). The difference in prevalence between both regions is predominantly visible in chest X-rays ordered by specialists (Table 3).

### 3.1. Occurrence of SPES

Of all 179 patients with pectus excavatum who belonged to the age group in which symptomatic pectus excavatum in seniors could occur, sufficient clinical data could be obtained for 117 patients. In these 117 patients, 38 (32.4%) had symptoms that might reflect the presence of symptomatic pectus excavatum in seniors with no other disorder mentioned in the patient's medical record that could be held responsible for these symptoms. The strength of the relation between symptoms and pectus excavatum is reflected in Table 4.

In our regression analysis, we tried to find what factor(s) would explain differences in the level to which patients may suffer from symptomatic pectus excavatum in seniors as expressed in the SPES sum score. We found no relation between the recorded symptoms of patients (SPES sum score) and the degree of the pectus excavatum as expressed in the Haller index (*P* = 0.30) nor did we find any relation between the SPES sum score and the patient's age (*P* = 0.29). We found, however, a significant relation between the SPES sum score and the radiological level of pectus excavatum (*F* = 3,45; *P* = 0.02).

## 4. Discussion

Our findings show that, apart from a prevalence of pectus excavatum in (at least) one in 400 patients referred for X-ray by primary care, a surprisingly high percentage has symptoms for which pectus excavatum could be held responsible. With this prevalence in mind and based on the reported prevalence of pectus excavatum in the population, among the Dutch senior population of four million people there would be 10.000 seniors with a pectus excavatum, of which one-third may have symptoms related to symptomatic pectus excavatum in seniors. Before drawing conclusions, a few points need further consideration.

This explorative study has undoubtedly suffered from underregistration. Pectus excavatum is presumably seen more often than it is reported. In several patients, pectus excavatum was reported only once in a series of 5–10 chest X-rays made in each individual patient. Mostly this concerned patients from specialist care. In the general practice population prevalence is, therefore, presumably higher than reported. Our prevalence data, however, are in line with data from other studies (which may have suffered from a similar underregistration). It is likely that the data from X-rays ordered by GPs are the most reliable as these contain the least patients with repeated chest X-rays.

Ideally, the study would have been set up as a case-control study. Unfortunately, this is not possible, mainly because we had no access to a comparable retrospective control group. While the presence of pectus excavatum may not be mentioned, it is even much more unlikely that the absence of pectus excavatum is not mentioned. Routine chest X-rays from an unscreened population are, therefore, unreliable. It should not, however, be forgotten that our study was basically meant as a first observation whether there could be any relation between pectus excavatum and the aforementioned symptoms.

Our findings suggest a relation between (the level of) pectus excavatum and symptoms such as shortness of breath and fatigue. The more prominent a pectus excavatum is on chest X-rays (as expressed in the level of pectus excavatum we assessed), the more likely it becomes that patients suffer from complaints. Typically, the Haller index that was developed 20 years ago clearly has no relation with symptomatic pectus excavatum in seniors. In our data, we found no clear relation with the degree in which patients have symptoms. This Haller index was developed to assess and quantify the level of pectus excavatum in children, and, therefore, it may not reflect the situation in adults.

In the literature, it is suggested to consider symptomatic pectus excavatum as a cardiovascular disorder [9, 10]. Our findings support this assumption. In daily practice, it can be envisioned that many SPES patients will have consulted a cardiologist and/or a pulmonologist, with often no overt explanation of their symptoms. Based on our findings, there is a reason to believe that pectus excavatum is not always as harmless as it seems [5]. Our data suggest a relation between pectus excavatum and symptoms like fatigue, shortness of breath, and palpitations. In the elderly especially, these symptoms may be confused with cardiac problems such as heart failure. This is especially relevant as in the case of symptomatic pectus excavatum in seniors there are treatment options allowing complete recovery. Unfortunately, concerning pectus excavatum and the possible symptoms, there is still much to explore. First of all, prospective research could focus on pectus excavatum and the risk of developing symptoms like dyspnoea, fatigue, and palpitations. Furthermore, research on the pretest probability of signs and symptoms is recommended. Further research is also needed on the effects of surgical repair of pectus excavatum. Two meta-analyses suggest effects of surgical repair, but the evidence is not as solid as one would wish [9, 11].

From a practical viewpoint, our findings suggest that a pectus excavatum found when examining the patient should not be neglected and should be considered as a possible explanation for symptoms like shortness of breath and/or palpitations (especially after exercise or when postural), chronic fatigue, and arrhythmia in the absence of an adequate other explanation.

## Figures and Tables

**Table 1:** Signs and symptoms of symptomatic pectus excavatum in seniors (SPES) [1–5].

Characteristics	
History	Shortness of breath, palpitations (after exercise or postural), and chronic fatigue
Physical examination	Deformity of the sternum
Electrocardiography	Ventricular extrasystoles and/or atrioventricular nodal tachycardia
Hypothesis	No adequate other explanation

**Table 2:** SPES score calculation.

Clinical findings	Score			
	0	1	2	3
Dyspnoea	Absent	Minor	Moderate	Severe
Palpitations	Absent	Minor	Severe	
Fatigue	Absent	Minor/moderate	Severe	
Chest pain	Absent	Yes		
Postural symptoms	Absent	Possible	Clearly present	
Pectus excavatum verified		Moderate	Severe	
ECG (supra)ventricular arrhythmia	Absent	Clearly present	Severe	
Echocardiography: mitral valve insufficiency and/or prolapse, tricuspid valve insufficiency, and enlarged right atrium	Normal	Minor	Clearly present	
Treadmill ECG	Normal	Stopped due to exhaustion		
Spirometry: inspiratory obstruction	Absent	Moderate	Clearly present	Severe

**Table 3:** Prevalence of pectus excavatum (PE) in an analysis of 159122 chest X-rays in the period of January 2004 until September 2007.

Ordered by	Atrium MC Heerlen			MUMC		
	X-rays	PE	Prevalence	X-rays	PE	Prevalence
GP	19048	45	0.24% (1 in 400)	14646	35	0.24% (1 in 400)
Cardiologist	23835	38	0.16% (1 in 600)	2871	5	0.17% (1 in 600)
Lung specialist	44764	18	0.04% (1 in 2500)	Unknown		
Others	45352	24	0.05% (1 in 2000)	8607	14	0.16% (1 in 600)
Overall	132999	125	0.09% (1 in 1000)	26124	54	0.21% (1 in 500)

**Table 4:** Relation between clinical assessment and radiological assessment (level of PE and Haller index, SPES sum scores categorised in 6 groups).

SPES sum score	*N*	Percentage of PE patients (*N* = 117)	Mean level PE	Mean Haller	SPES
>8	8	6.8%	4.0	2.8	Almost certain
7 or 8	15	12.8%	3.8	2.8	Likely
5 or 6	15	12.8%	2.9	3.0	Possible
3 or 4	24	20.5%	3.0	2.8	Not likely
1 or 2	30	25.7%	3.0	3.1	Not present
0	25	21.4%	3.0	2.8	Not applicable
